# ﻿Taxonomic note on the species status of *Epiophlebiadiana* (Insecta, Odonata, Epiophlebiidae), including remarks on biogeography and possible species distribution

**DOI:** 10.3897/zookeys.1127.83240

**Published:** 2022-11-02

**Authors:** Sebastian Büsse, Jessica L. Ware

**Affiliations:** 1 Functional Morphology and Biomechanics, Institute of Zoology, Kiel University, Am Botanischen Garten 9, 24118 Kiel, Germany Kiel University Kiel Germany; 2 Division of Invertebrate Zoology, American Museum of Natural History, 200 Central Park West, New York, NY 10024, USA American Museum of Natural History New York United States of America

**Keywords:** Anisozygoptera, *Epiophlebialaidlawi*, *E.sinensis*, *E.superstes*, genetic sequences, relict dragonfly, synonymy

## Abstract

The species included in the genus *Epiophlebia* Calvert, 1903 represent an exception within Recent lineages – they do not belong to either dragonflies (Anisoptera) nor damselflies (Zygoptera). Nowadays, the genus is solely known from the Asian continent. Due to their stenoecious lifestyle, representatives of *Epiophlebia* are found in often very small relict populations in Nepal, Bhutan, India, Vietnam, China, North Korea, and Japan. We here present a taxonomic re-evaluation on the species status of *Epiophlebiadiana* Carle, 2012, known from the Sichuan province in China, supplemented with a morphological character mapping on a genetic tree to highlight synapomorphies of *E.diana* and *E.laidlawi* Tillyard, 1921. We conclude that *E.diana* is a junior synonym of *E.laidlawi*. Furthermore, we discuss the Recent distribution of the group, allowing for predictions of new habitats of representatives of this group.

## ﻿Introduction

Odonata Fabricius, 1793 are classified into the suborders Anisoptera Sélys, 1854 (dragonflies), Zygoptera Sélys, 1854 (damselflies), and the enigmatic taxon, *Epiophlebia* Calvert, 1903. Presently, the genus *Epiophlebia* is considered to be the sister-group of the Anisoptera [Anisoptera + *Epiophlebia* = Epiprocta Lohmann, 1996], with several extinct lineages nested in between (cf. Bechly 1996; Lohmann 1996; Rehn 2003; Fleck et al. 2003; Grimaldi and Engel 2005); the validity of Epiprocta is supported by numerous phylogenetic studies (cf. Hovmöller et al. 2002; Fleck et al. 2008; Bybee et al. 2008, 2021; Blanke et al. 2012, 2013; Misof et al. 2014; Letsch et al. 2016; Büsse et al. 2018; Kohli et al. 2021; Suarov et al. 2021). These taxa were considered to form a suborder, called ‘‘Anisozygoptera’’, which comprise mainly Jurassic fossils (Nel 1993) and the recent species of the genus *Epiophlebia*, until it was shown that ‘‘Anisozygoptera’’ are polyphyletic (Nel 1993; Lohmann 1996; Rehn 2003). Because the species of the genus *Epiophlebia* show some distinct characters of Zygoptera as well as Anisoptera (Asahina 1954; Büsse 2016), they are often considered as relict species (Asahina 1954; Davies 1992; Mahato 1993). From a morphological point of view, the genus *Epiophlebia* seems to represent the most ancestral character distribution of Recent Odonata (Blanke et al. 2012, 2015; Büsse et al. 2015; Büsse 2016).

**Figure 1.**
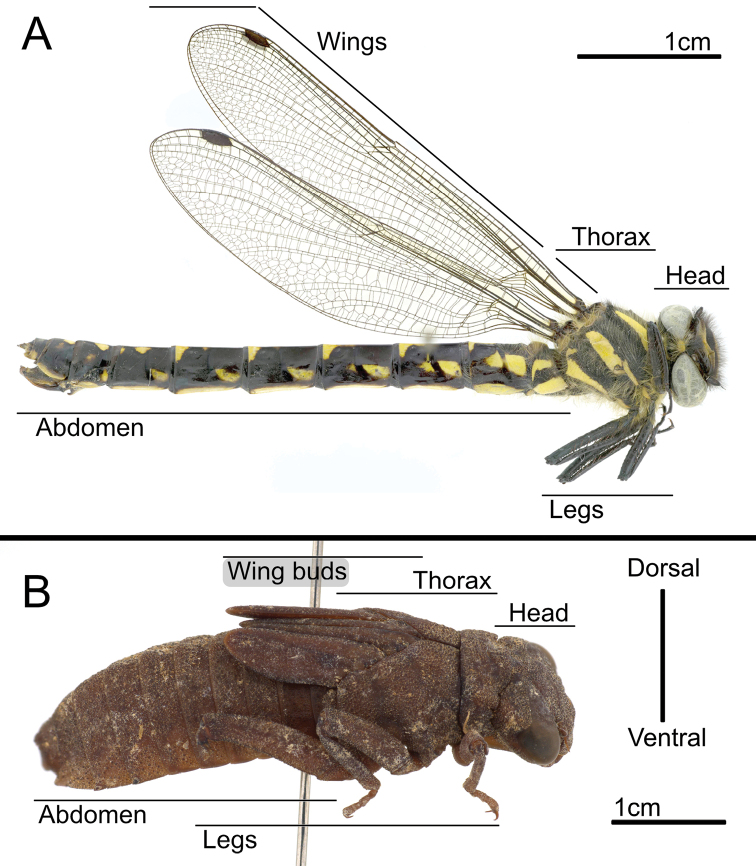
*Epiophlebiasuperstes* from Japan, lateral view **A** adult **B** larva.

Adult *Epiophlebia* are very conspicuous (Fig. 1), and in the field they can easily be identified by the black-yellow striped coloration (Asahina 1954) and their characteristic slow and rather uncoordinated appearing undulating flight (Rüppell and Hilfert 1993). Morphologically, the anisopterous body shape, the zygopterous shape of the wings, and the convex frons are some of the main distinguishing characteristics (Asahina 1954; Büsse 2016). The larvae of *Epiophlebia* also resemble dragonflies, as they use a rectal chamber for respiration, but jet propulsion, which is typical for Anisoptera (Corbet 1999), has never been observed (Tabaru 1984). Their morphological distinction is rather subtle, so they are easily mixed up with, for example, gomphids or petalurids (Asahina 1954) – as happened to *Epiophlebiadiana* Carle, 2012. The type specimens of *E.diana* were collected by “Dr. David C. Graham in the mountainous regions of western Szechuan” (Needham 1930). Needham however, misidentified the larvae of *Epiophlebia* as Gomphidae (Carle 2012).

While the ancestors of present *Epiophlebia* species were at their peak in the Mesozoic era and were possibly distributed over large areas on the pre-Asian continent (Carpenter 1992; Nel et al.1993), recent species have restricted ranges, often confined to small areas in Asia: *Epiophlebiasuperstes* Selys, 1889 in Japan; *Epiophlebialaidlawi* Tillyard, 1921 in Nepal, India, Bhutan, and Vietnam; *Epiophlebiasinensis* Li & Nel, 2012 in North Korea and China, and *Epiophlebiadiana* also in China (Asahina 1954; 1963; Tani and Miyatake 1979; Büsse et al. 2012; Carle 2012; Li et al. 2012; Fleck et al. 2013; Büsse 2016) showing a characteristic disjunct distribution (Büsse et al. 2012).

Since the habitat requirements of the genus *Epiophlebia* seem to be very specific, the range of Recent habitats is extremely restricted. *Epiophlebia* species prefer cold mountain streams with temperatures of about 4 to 5 °C in winter and about 16–17 °C in summer (data published for *E.superstes* by Tabaru (1984)) and altitudes between 1,300 to approximately 3,000 m (data published for *E.laidlawi* by Brockhaus and Hartmann (2009)). This stenoecious lifestyle has restricted the genus *Epiophlebia* to cold habitats, like glacial refuges (De Lattin 1967; Büsse et al. 2012).

For recently diverged species, or for taxa that are described under the assumption of incipient speciation, it can be challenging to develop a morphological character set that reveals the true pattern of evolutionary history for a taxon. Further complicating matters is that there are often separate, not cross-referenced, descriptions of adults and larvae for Odonata. In the case of the genus *Epiophlebia*, adults and larvae are described for *E.superstes* and *E.laidlawi*, while for *E.sinensis* only the adults and for *E.diana* only the larva is known. The species status of *E.diana* has already been critically discussed and is doubtful (cf. Dijkstra et al. 2013; Büsse 2016). We, therefore, present a taxonomic re-evaluation of the species status of *E.diana*. Unfortunately, the type specimen is untraceable and seems lost (F.L. Carle, author of *E.diana* as well as J.J. Dombroskie of the Cornell University Insect Collection, New York, USA: personal communication, see Büsse 2016). However, a combination of morphology, phylogeny, and biogeography, described here, lays a solid basis for the designation of *E.diana* as junior synonym of *E.laidlawi*.

## ﻿Materials and methods

**Figure 2.**
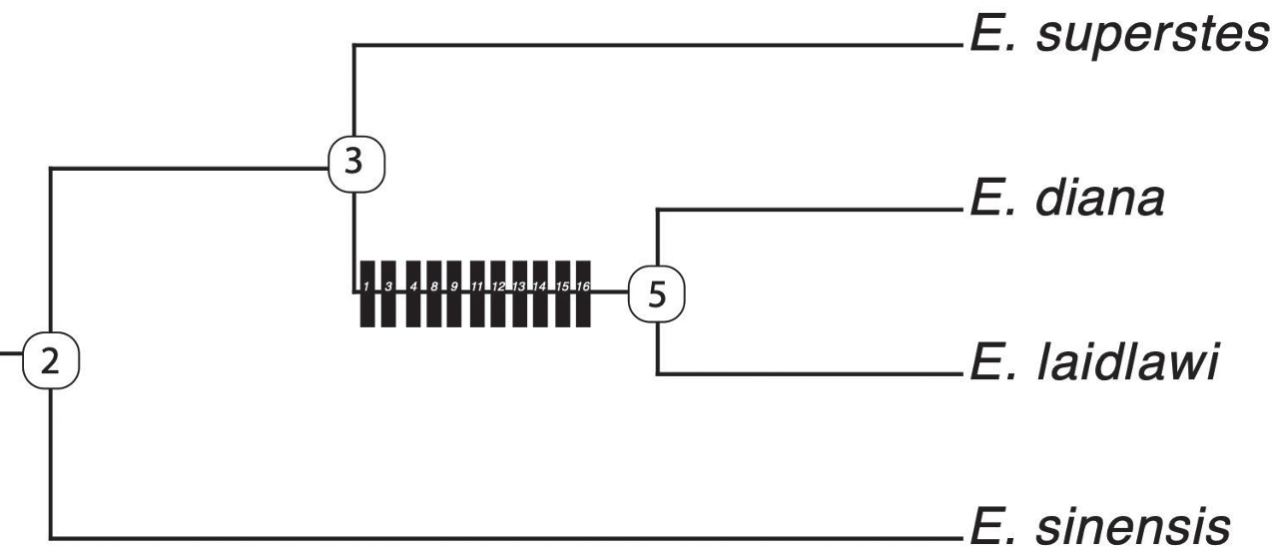
Character mapping on a strict consensus tree based on Büsse et al. (2012). Synapomorphies are shown as black boxes, numbers indicate which of the characters shown in Table 1 serves as the synapomorphy.

Here, we examined morphological data from Büsse (2016) and several other publications (i.e., Asahina 1954, 1961; Büsse et al. 2012; Carle 2012; Li et al. 2012; Dorji 2015). A matrix composed of all characters used in the past to evaluate *Epiophlebia* larval and adult characters is shown in Table 1; briefly this includes larval traits and adult characters related to size and colouration, features of the head, abdomen, genitalia, and appendages. The phylogeny that was used here was based on Büsse et al. (2012; fig. 2). The main justification for using Büsse’s and colleagues (2012) phylogeny is that presently there are few overlapping sequence fragments across the species of *Epiophlebia*. Here, we examined all available mitochondrial and nuclear gene fragments for the genus *Epiophlebia* from GenBank to evaluate what available data was present for the genus (Table 2). Unfortunately, to date *E.superstes* is the only species for which sequence data are available for a broad sampling of genes. No genetic data is available for *E.diana*, presumably because the describing author has misplaced the existing specimens (F.L. Carle personal communication). With such a dataset, we decided to use the phylogeny provided by Büsse et al. (2012) which is the most comprehensively sampled phylogeny for the genus currently available. In terms of character mapping, briefly, characters were traced in Mesquite (Maddison and Maddison 2016) using both the ancestral state reconstruction parsimony and likelihood functions. Consistency index values for a matrix including all traits in Table 1 were evaluated in Mesquite against a tree assuming (*E.sinensis* (*E.superstes* (*E.diana*, *E.laidlawi*))) and found to be 1.0.

**Table 1.** Morphological matrix for larvae and adults for key differences between *Epiophlebia* species (character states).

	Character	*E.superstes*	*E.laidlawi*	*E.diana*	*E.sinensis*
**Larvae**					
1	General colouration: 0 = darker, 1 = lighter (Büsse 2016), (Carle 2012), (Dorji 2015)	0	1	1	?
2	Scape and pedicle: 0 = scape and pedicle same length as flagellomere or shorter 1 = scape and pedicle always longer than first flagellomere (Büsse 2016), Carle 2012	0	{01}	1	?
3	Flagellomere: 0 = maximally as long as the 2^nd^ and 3^rd^ together or shorter, 1 = first longer than the 2^nd^ and 3^rd^ together (Büsse 2016), Carle 2012	0	1	1	?
4	Premental cleft: 0 = not distinctly developed, 1 = distinctly developed (Büsse 2016), Carle 2012	0	1	1	?
5	Spearhead-like processes on notum: 0 = not so, 1 = depressed posterolaterally (Büsse 2016)	0	1	?	?
6	Anterior ridge of the metathoracic post sternum: 0 = shallow, 1 = deep, cone like (Büsse 2016)	1	0	?	?
7	Abdominal stridulatory file of segment 7: 0 = well developed, 1 = vestigial on segment 3 (Büsse 2016), Carle 2012	0	[01}	1	?
8	Dorso-lateral edges abdominal segments 7–9: 0 = protruding and pointed, 1 = rounded (Büsse 2016), Carle 2012	0	1	1	?
9	Lateral abdominal lobes on segment 9: 0 = not very sinuous margins, not much protruding by lobes on segment 9, 1 = sinuous margins, lobes protrude on segment 9 (Büsse 2016), Carle 2012	0	1	1	?
10	Apices of the epiproct: 0 = divided distinctly, 1 = divided slightly (Büsse 2016)	1	0	?	?
**Adult**					
11	Adult abdomen colour: 0 = blackish with more yellow markings; 1 = brownish with less yellow markings (Asahina 1954; 1961) (Büsse 2016), (Dorji 2015) (Li et al., 2012)	0	1	?	0
12	Adult abdomen segments 2–7with yellow spot on posterior margins: 0 = no, 1 = yes (Asahina 1954; 1961) (Li et al., 2012)	0	1	?	0
13	Adult thorax with 2 narrow yellow lateral stripes: 0 = no, 1 = yes (Asahina 1954; 1961) (Li et al., 2012)	0	1	?	0
14	Forewing and Hindwing light yellow brownish: 0 = hyaline, 1 = light yellow brownish wings (Asahina 1954; 1961) (Li et al., 2012)	0	1	?	0
15	Abdomen with dorsal stripes: 0 = no, 1 = yes, (Asahina 1954; 1961) (Li et al., 2012)	1	0	?	1
16	Abdomen segment 10: 0 = mainly black with yellow lateral spots, 1 = not so (Asahina 1954; 1961) (Li et al., 2012)	1	0	?	1

**Table 2.** Available mitochondrial and nuclear gene fragments for *Epiophlebia* species in GenBank.

	COI	COII	12S & 16S	Complete mitochondrial genome	18S & 28S	Elongation Factor alpha	Opsin fragments	Histone 3	ITS1 & ITS2
*Epiophlebiadiana*	–	–	–	–	–	–	–	–	
*Epiophlebialaidlawi*	–	2 sequences	–	–	6 sequences	–	–	–	3 sequences
*Epiophlebiasinensis*	–	–	–	–	–	–	–	–	2 sequences
*Epiophlebiasuperstes*	19 sequences	1 sequence	23 sequences	2 sequences	24 sequences	1 sequence	24 sequences	1 sequence	2 sequences

For photography, we used specimens of *E.superstes* (because of availability) to depict the general habitus of the very similar *Epiophlebia* species. For stacked photography, a custom-made 3D-printed illumination dome system (Bäumler et al. 2020) and an Olympus OMD 10mkII digital camera (Olympus K.K., Tokyo, Japan), equipped with a Leica 45 mm macro lens (Leica Camera AG, Wetzlar, Germany) was used. All images were subsequently processed in Affinity Photo and Affinity Designer (Serif Ltd, Nottingham, United Kingdom).

## ﻿Results and discussion

### ﻿Taxonomy

A comparison of the morphological characters used in past studies to the currently accepted phylogeny of the genus *Epiophlebia* suggests that several characters are not useful for reconstruction of the evolutionary history, as they are only known for adults of all species except *E.diana*, or only known for larvae of all species except *E.sinensis*. Using parsimony, we found 10 characters supporting a clade comprising *E.laidlawi* and *E.diana* (Fig. 2), but as five of those characters are based on adult traits, there are missing data for *E.diana*. Furthermore, for several distinguishing characters employed by Carle (2012), the reported characters of *E.diana* fall within the trait range reported for *E.laidlawi*, while some characters even seemed to be poorly scored by Carle (2012). For example, he described the abdominal stridulatory files (ASF) in the genus *Epiophlebia*. He mentioned for *E.superstes* that the ASF of segment 3 is well developed, and the ASF segment 4 is about as high as long, and the ASF segment 7 is vestigial. In the data of Büsse (2016), specimens of *E.superstes* can be found with almost no stridulatory file on segment 3, and the ASF of segment 4 all can be seen higher as long, shorter as long, and as long as high. Furthermore, Carle (2012) suggested for the distinction of *E.diana* differences in the ASF (for *E.diana* he listed ASF of segment 7 c. 3/4 length of segment and for *E.laidlawi* the ASF of segment 7 c. 1/2 length of segment), but these are not valid as there are *E.laidlawi* in the data showing c. 3/4 as well (Büsse 2016). Next, Carle (2012) listed distinctions between the two species based on the degree of sinuity in the premental margins (in *E.laidlawi*, prementum with lateral margins slightly sinuous, but in *E.diana*, prementum with lateral margins strongly sinuous). It is difficult to estimate what slightly and strongly means, as such wording is subjective in nature; other points of view may consider the lateral margins of the prementum in *E.laidlawi* to be not just slightly sinuous, and without a figure showing data from Carle, it is impossible to say whether the sinuous nature of the prementum in *E.diana* is more pronounced; this character is not diagnostic. Similarly, Carle (2012) listed the fore-femur as being c. 3.0× as long as wide in *E.laidlawi* and for *E.diana* 2.5× as long as wide; this character is not valid for distinction between these species as there is variation in this trait and *E.laidlawi* have been documented with fore-femur that are only 2× as long as wide and there are *E.superstes* in the data showing a fore-femur c. 3× as long as wide (Büsse 2016). In fact, Asahina (1961) noted as the distinguishing character of *E.laidlawi* and *E.superstes* that the fore-femur of *E.laidlawi* was longer. It seems this character is very variable and impractical for taxonomic use. Indeed, fore-femur length has been shown to be influenced by ontogeny, and it is rarely used to infer evolutionary history. Lastly, Carle (2012) listed *E.laidlawi* with lateral abdominal lobes slightly protruding on segment 9 and *E.diana* with lateral abdominal lobes protruding on segment 9. Again, these are subjective descriptions, and Büsse’s (2016) data show abdominal lobes slightly protruding on segment 9 in *E.superstes* and distinctively produdent, forming a distinctive overhanging protrusion at the end of the segment 9 compared to the preceding segments, in *E.laidlawi*, comparable to Carle’s (2012) fig. 3D. Only younger larvae of *E.laidlawi* seem to have only slight protudents on segment 9; here the abdominal segments resemble each other comparable to Carle’s (2012) fig. 2D. In short, although no specimens of *E.diana* are available to examine, the characters used by Carle (2012) to describe the species do not seem to show a bimodal distribution of character values between *E.diana* and *E.laidlawi*, and given known and documented phenotypic variation in these traits for *E.laidlawi*, we consider *E.diana* a synonym of *E.laidlawi*.

**Figure 3.**
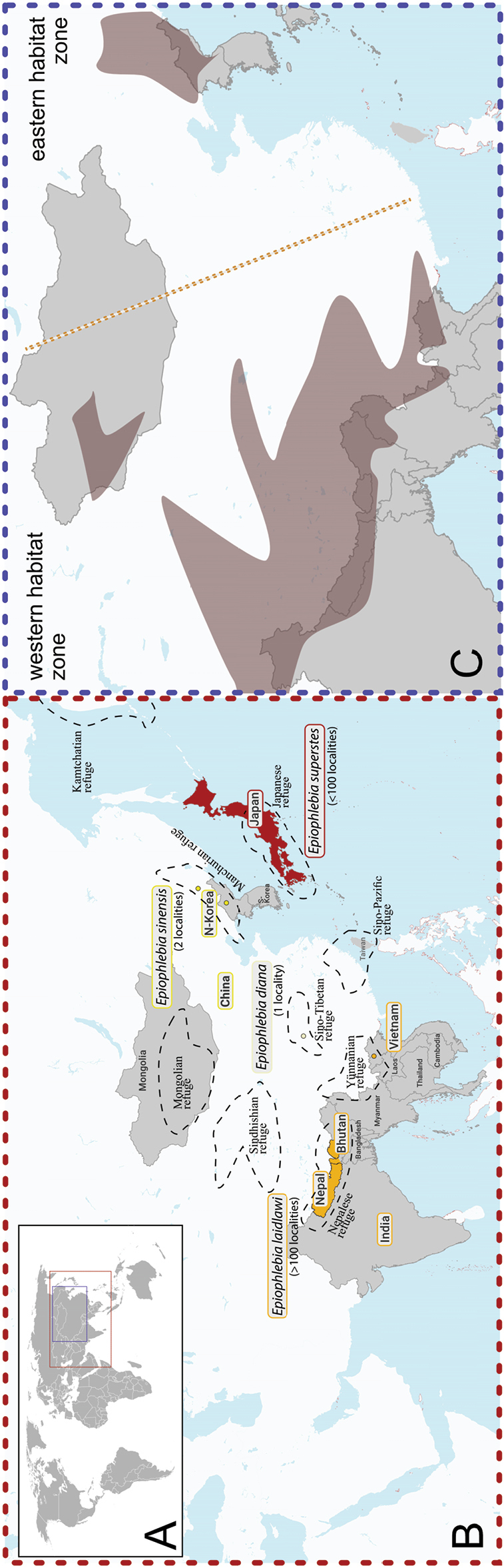
Maps of Asia (excerpt) **A** overview map, indicating map excerption of **B** red square and **C** blue square **B** known distribution of *Epiophlebia* species in: Bhutan, China, India, Japan, Nepal, North Korea, and Vietnam, and including glacial refuges after De Lattin (1967)**C** simplified mountain regions (brownish shadings) of the Asian mainland. Illustrating the large portions of temperate Chinese lowlands separating the western and eastern *Epiophlebia* habitat zones, clearly showing the affiliation of *E.diana* to the western (*E.laidlawi*) habitat zone.

### ﻿Biogeography

The described stenoecious lifestyle has restricted the genus of *Epiophlebia* to cold habitats, indicated by the recent distribution in glacial refuges (Fig. 3B; De Lattin 1967; Büsse et al. 2012) – *E.superstes* from the Japanese refuge, *E.laidlawi* from the Nepalese and the Yunnanian refuges, *E.sinensis* from the Manchurian refuge (more precisely Ussurian secondary centre), and ‘*E.diana*’ from the Sino-Tibetan refuge – thus, clearly showing a separation in a western and eastern habitat zone (Fig. 3C). Due to the distribution in the mentioned glacial refuges, we predict that other *Epiophlebia* habitats may exist in the Sino-Pacific refuge, the Sindhisian refuge, the Mongolian refuge, and further populations in the Manchurian refuge because it is divided into secondary refuges, as well as the Kamtchatian refuge. Whether one can expect new species of *Epiophlebia* or new populations of a known species in these possible habitats is to be answered.

Indeed, the connection between Japan and the Asian mainland, as well as regions of the Himalayas and other parts of Asia, has been well documented by Ikeda and Ohba (1998) and is known as the Sino-Japanese floristic region during the last ice ages (Ikeda and Ohba 1998; Büsse et al. 2012). The question remains as to when the extant *Epiophlebia* species diverged, as two contradicting hypotheses are plausible: i) *Epiophlebia* dates back to the Jurassic when Pangaea broke apart (Brockhaus and Hartmann 2009), or ii) *Epiophlebia* diverged during to the last or second last ice age period (Büsse et al. 2012; Büsse 2016). To substantiate one of these biogeographic scenarios, a re-analysis is absolutely necessary.

Nowadays, the habitats of *Epiophlebia* species are widely separated. Japan is separated from the mainland by sea-straits with depths of approximately 55 m north of Hokkaido and 130 m between the southern island of Kyushu and Korea (Millien-Parra and Jaeger 1999). In addition to the ocean, there are approximately 3000 km (respectively more than 3600 km) of temperate lowlands separating Japan, inhabited by *E.superstes* and the known ranges of *E.laidlawi* and ‘*E.diana*’. The same is true for the habitat of *E.sinensis* in Heilongjiang province, China (Li et al. 2012), as it is more than 3000 km away from the cold mountain habitat of *E.laidlawi* in the Himalayas and separated by temperate lowlands. The eastern and western habitat zones are, thus, separated by unsuitable, temperate lowlands (Fig. 3C). The location where the synonymized ‘*Epiophlebiadiana*’ was found in Sichuan province, China (Carle 2012), is also part of the western habitat zone, as the known range of *E.laidlawi*. The known distributions of ‘*E.diana*’ and *E.laidlawi* are around 1000 km apart but are connected by the mountain range of the Himalayas, which contains ample suitable habitats for an *Epiophlebia* species.
